# A New Self-Activated Micropumping Mechanism Capable of Continuous-Flow and Real-Time PCR Amplification Inside 3D Spiral Microreactor

**DOI:** 10.3390/mi10100685

**Published:** 2019-10-11

**Authors:** Kangning Wang, Di Wu, Wenming Wu

**Affiliations:** Changchun Institute of Optics, Fine Mechanics and Physics (CIOMP), Chinese Academy of Sciences, Beijing 100864, China; 15098139057@163.com (K.W.); wudi16@mails.ucas.ac.cn (D.W.)

**Keywords:** microfluidics, viscosity, continuous flow, real-time PCR

## Abstract

A self-activated micropump which is capable of stable velocity transport for a liquid to flow a given distance inside a 3D microchannel has been a dream of microfluidic scientists for a long time. A new self-activated pumping mechanism has been proposed in this paper. It is different from the authors’ previous research which relied on the fluid resistance of a quartz capillary tube or end-blocked gas-permeable silicone or a polydimethylsiloxane (PDMS) wall to automate the flow. In this research, an end-open stretched Teflon tube was utilized for passive transport for the first time. A new fluid transmission mode was adopted with the assistance of a cheaper easily accessible oil mixture to achieve stable continuous flow. Finally, this novel micropump has been applied to real-time continuous-flow polymerase chain reactions (PCRs), with an amplification efficiency similar to that of a commercial PCR cycler instrument.

## 1. Introduction

As the “heart” of a microfluidic system, micropumps play a significant role in fluidic transport. Due to the important functions of micropumps, many different principles have been designed to automate the transportation of a fluid [1,2,3]. Depending on the power source, all micropumps can be divided into two categories: Externally-powered and self-powered micropumps. Additionally, all these micropumps have huge application prospects in a wide range of fields, such as drug delivery, blood transport, chemical and biological analysis, and electronic cooling [4,5].

Several types of self-powered micropumps have been developed over the last few years. These self-powered micropumps normally use gas diffusion/permeation [6,7,8], surface tension [9,10], hydrostatic force [11,12], evaporation pressure [13], or electrical forces [14,15,16] to induce the fluid flow in microfluidic devices.

A self-powered micropump which depends on the permeability coefficient of a silicone [17] or polydimethylsiloxane (PDMS) elastomer [10,18] to control its flow velocity has been proposed. Although this type of passive micropump has displayed good performance for stable sample transport, there are non-negligible shortcomings associated with this method. In the past few years, to achieve a stable flow velocity inside long microchannel during long term, a self-powered micropump that depends on the permeability coefficient of the silicone or PDMS elastomer to control the flow rate was proposed [17,18]. Because this type of micropump has an advantageous performance for stable liquid transport, even inside long microchannel concerning 3D channel-configuration and high temperature condition, there is a non-negligible shortcoming that the self-powered flow is virtually automated by the gas permeability of the silicone [17] or PDMS wall [18], but the specified permeability coefficient of silicone or PDMS is determined by the size distribution of mini-pores in the elastomer, which usually varies from the fabrication process, curing conditions, and the production batch, and thus increases the difficulty in accurately controlling the passive flow rate.

Recently, the authors have introduced a novel pumping principle and method for application in both the passive and stable velocity transport of a liquid [19,20]. This was achieved by applying an end-open gas-impermeable quartz tube to automate the flow for the first time. Although the pump benefitted from this improvement, the flow velocity was fixed for the same pressure, as long as the inner diameter and the length of the quartz tube was fixed. So, there is no need to worry about the variance of the permeability coefficient of silicone or a PDMS elastomer which can be easily influenced by differences in the fabrication process. However, this method still has some non-negligible disadvantages. Firstly, the inner diameter of the tail quartz tube is too small to be able to let the liquid flow smoothly; so, when the liquid enters the quartz tube, there is a great possibility that it will jam the tube and influence the flow velocity of the latter part of the liquid plug. Secondly, the size of the quartz tube is limited and can only be obtained with set inner diameter sizes such as 10 µm, 25 µm, and 50 µm. This means that it is difficult to precisely control the flow and achieve the desired results.

A new method of stretching the Teflon tube at the end of the system to four times its original length to achieve precise control of the flow velocity has been proposed in this paper. This method has not only inherited the advantages of the previous methods, but it also avoids their shortcomings. It is known from Hagen-Poiseuille’s law that when a 15 cm-long Teflon tube (ID = 0.3 mm, OD = 0.6 mm) is stretched to four times its original length, the flow resistance that it causes is the same as that of a 6 cm-long quartz tube (ID = 25 µm). The purpose of using stretched tube instead of quartz tube is to solve the problem that the fluid velocity changes greatly after the liquid enters the smaller diameter quartz tube (ID = 25 µm). Previous experiments showed that when liquid with different viscosities flows to the entrance of the quartz tube, the flow rate changes greatly [19,20]. The reason for this phenomenon is that the inner diameter of the interface between quartz tube and Teflon tube has changed dramatically, due to a sudden great change of the resistance at the junction. In order to ensure that the flow velocity of the fluid inside the Teflon tube with different viscosities does not change when they flow into tubes with smaller diameters, the method of stretching the original tubes is adopted, which can not only provide the fluid resistance that the quartz tube do, but also make the inner diameter of pipeline change continuously instead of suddenly becoming smaller. In this way, the problem of fluid velocity instability in the Teflon tube caused by different viscous liquids entering the tube could be solved.

Therefore, a series of experiments were designed to verify the feasibility of this method by exploring the effect of a liquid’s viscosity and pressure on flow control, and the system was applied to both continuous-flow and real-time polymerase chain reactions (PCRs). The results that have been demonstrated in this paper showed that the proposed method is totally feasible, and it can not only achieve steady flow control of a liquid, but the design of the end of the system is flexible enough to realize any flow velocity requirements under almost any conditions.

## 2. Principle

**Figure 1:**
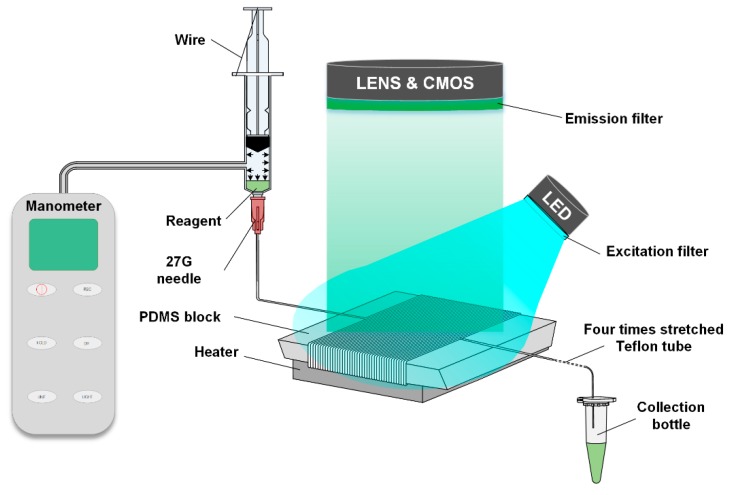
Principle of the end-opened system.

Figure 1 has shown a diagram of the actuation mechanisms of the end-open self-activated micropump system. The system is based on the air permeability from the fluidic conduit to the atmosphere. Since the pressure of the compressed air inside the fluidic conduit is much higher than the external atmospheric pressure, the air molecules inside the channel tend to penetrate through the hollow channel of the stretched Teflon tube to the outside atmosphere. The permeation of the air only occurs at the site of the outlet of the Teflon tube which causes a decrease of the air molecules’ mole-number in the anterior end of the sample plug. This can be calculated using the following equations:

(1) $$G_{a}=\frac{DA_{ave}}{Z}\left(C_{Aia}-C_{Ao}\right)$$

(2) $$C_{Aia}=\frac{P_{a}}{RT}$$

(3) $$C_{Ao}=\frac{P_{Ao}}{RT}$$

(4) $$A_{ave}=a\pi r_{q}^{2}$$

(5) $$P_{a}=P_{p}-P_{g}$$

where $G_{a}$ is diffusion rate, D is equivalent diffusion coefficient, $C_{Aia}$ is inner air molecule concentration in the anterior end of sample plug, $P_{a}$ represents the air pressures in the anterior end of the reagent, $C_{Ao}$ is air molecule concentration of ambient atmosphere, Z is diffusion distance and can be represented by the length of the stretched Teflon tube, $A_{ave}$ is the average diffusion area, $r_{q}$ is the diameter of the stretched Teflon tube, a is the correction coefficient between actual condition and an ideal condition. $P_{p}$ is the air pressures in the posterior end of the reagent, $P_{g}$ is the pressure gradient imposed in the reagent.

It can be regarded as an equivalent condition when the fluidic-flux and air-molecule-penetration is the same with each other, producing the constant pressure $P_{a}.$

Suppose the microchannel is column-configuration, then

(6) $$v=\frac{Dar_{q}^{2}}{RTr^{2}}\frac{dP}{dZ}$$

where v is the velocity of the microfluidic, r is the radius of the microchannel, dP/dZ is the pressure gradient across the hollow channel of the stretched Teflon tube connecting the microchip to the atmosphere. Easily seen, the flow rate decreases if the radius of the microchannel increases.

Besides, it is known from Hagen-Poiseuille’s law which can be written as follows [21]:

(7) $$P_{r}=8\mu l\frac{u_{i}}{R^{2}}$$

where $P_{r}$ is the pressure difference, *R* is the inside radius of the tail Teflon tube, *µ* is the viscosity coefficient of the liquid, *l* is the length of the tail Teflon tube and u is the flow velocity. Therefore, it can be calculated that when a 15 cm-long Teflon tube (ID = 0.3 mm, OD = 0.6 mm) is stretched to four times its original length, the flow resistance of the tube will be the same as a 6 cm-long quartz tube (ID = 25 µm).

What is more, an experiment was designed by the authors to test the effect of both viscosity and pressure on the flow velocity. Based on Equation (7) it can be known that the flow velocity increases as the viscosity of the oil mixture decreases and that the flow velocity increases as the pressure increases [22,23].

It is noteworthy that the principle we proposed applies only to ideal gases, and the experimental results may be slightly different from the theoretical predictions. Besides, the influencing factors of fluid flow at nanoscale, such as the Darcy-Weisbach friction factor [24,25], only has negligible influence on our experiment, since the inner and outer diameters of the Teflon tubes we used are 0.3 mm and 0.6 mm, respectively. After being four times stretched, the inner and outer diameters of the stretched Teflon tubes are 0.16 mm and 0.32 mm, respectively. This showed that the size of our Teflon tubes did not reach nanoscale, but micron-scale. So, the phenomenon will not be taken into account in this paper.

## 3. Experimentation 

### 3.1. Flow Experiment

As shown in Figure 1, a system was assembled to verify the performance of the liquid transportation. During the assembly of the system, the Teflon tube (ID = 0.3 mm, OD = 0.6 mm) was wrapped around a PDMS block 40 times. The PDMS block was fabricated using a cooper mold with a trapezoidal cross section, and the widths of the top and the bottom surfaces were 35 mm and 10 mm, with the height and length set to be 13.5 mm and 50 mm, respectively. Because a trapezoidal shape block can easily achieve the proper reaction time for PCR reaction under a constant flow speed. Approximately 15 cm of the Teflon tube was saved at the end of the system and then stretched to four times its original length to 60 cm (ID = 0.16 mm, OD = 0.32 mm). The purpose of using PDMS was because it does not have a good thermal conductivity, so that we can achieve the optimum reaction temperature of the PCR reagent at a lower altitude. The advantage is that the distance between a high and low temperature cycle of the PCR reagent decreases, and the total flow time of the whole PCR reaction decreases.

During the flow assays, the device set-up consisted of a 10 ml syringe, a 27 G needle, and an iron wire. Then, 100 µL of a castor oil mixture, 20 µL of reagent and 900 µL of a castor oil mixture were sequentially added to the 27 G needle. The system was then activated by depressing the plunger of the syringe to a certain point, which was then fixed using the iron wire. During this procedure, the Teflon tubing is connected to the syringe. And the end of the tubing is not blocked during the depression of the plunger. Before connecting the syringe and the Teflon tube, the tube is filled with oil, then a small dose of the oil phase is added to the syringe, and a certain amount of reagent is injected into the syringe. After these procedures, the syringe and the Teflon tube are connected. After the system is assembled, the syringe is compressed and fixed with iron wire, and the liquid flow is driven by the pressure difference between inside and outside of the system.

Two groups of experiments were then designed to explore the flow of the liquid plug under different conditions.

1. Experiment on changing the viscosity of the oil phase

The experiment was carried out using three different proportions of the castor oil and isopropyl palmitate mixture which had different viscosities. In order to maintain the other control parameters at the same values, the temperature was set at 30 °C, and the pressure was set at 71.6 kPa. Each experiment was then carried out three times. In order to ascertain the relationship between the mixing ratio and the viscosity, a viscometer (LICHEN TECH, NDJ-1, Zhejiang, China) was used to measure the parameters. The experimental results have been shown in Table 1.

2. Experiment on changing the pressure in the syringe

The experiment was carried out with three different pressure values. In order to maintain the other control parameters at the same values, the temperature was set at 30 °C, and the mixing ratio of the oil mixture was 40%, which meant the viscosity was 21.7 mPa·s. Each experiment was then carried out three times.

### 3.2. Application in Continuous-Flow on-Chip PCRs

After the aforementioned flow experiments, the system was tested for application in continuous-flow on-chip PCRs. The pressure in the syringe was set at 71.6 kPa by adjusting the piezometer. The mixing ratio of the oil mixture was 40%, and all the connections were sealed using silicone adhesive.

**Figure 2:**
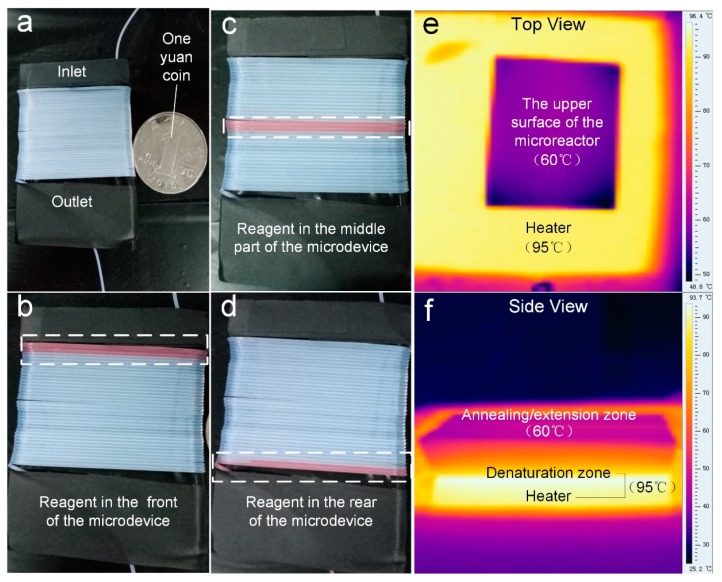
The set-up for continuous-flow on-chip PCR (**a**) with the red-color sample flowing at the start (**b**), the middle (**c**), and the end (**d**) of the fluidic conduit. (**e**) The top view and (**f**) the side view of the thermal infrared image of the trapezoidal PDMS block.

As for temperature control, a single heater was used to achieve the thermal cycle requirements by controlling the temperature gradient of the PDMS block. In order to make sure the temperatures of the upper and lower surfaces were suitable for continuous flow microfluidic PCRs, an infrared (IR) camera (Fotric 220, ZXF Laboratory, Dallas, TX, USA) was used to monitor the temperature of the PDMS block. The tube wrapped around PDMS block was placed on the top of the 95 °C heater (JXMINI-80, Jingxin Tech, Shanghai, China). As shown in Figure 2, the result showed that the entire PDMS block was able to reach a temperature that was suitable for the PCR reaction.

In order to prove that the system could be successfully used for continuous flow PCRs, a commercial PCR cycler (CFX Connect, Bio Rad, Hercules, CA, USA) was taken as a reference. By comparing the results of the two devices, the function of this system could be verified. 

The DNA fragment of the pGEM-3Zf (+) plasmid was amplified using the microreactor. The primer sequences for amplifying the gene fragment of pGEM-3Zf (+) were as follows: 5′ CCA GTC GGG AAA CCT GTC GTG CC 3′ (forward) and 5′ GTG AGC GAG GAA GCG GAA GAG CG 3′ (reverse). The PCR reagent was composed of 1× SYBR Premix Ex TaqII, 0.075 U µL^−1^ TaKaRa EX Taq, 0.6 mg mL^−1^ BSA (AS25483; AMEKO, Dalian, China), 1 µM of forward and reverse primers, and 0.03012 ng·µL^−1^ of template. Each test requires 20 µL of reagent.

### 3.3. Real-Time Fluorescence Detection

By comparing the intensity of the fluorescence and the products resulting from the commercial PCR cycler and the microdevice, we can prove that the system could be used for real-time continuous flow PCRs. 

The DNA fragment of the H7N9 was amplified using the microreactor. The primer sequences for amplifying the gene fragment of H7N9 were as follows: 5′ TAC AGA CAA TCC CCG ACC GA 3′ (forward) and 5′ GCC AAG TGT TAG CCC CAT CC 3′ (reverse). The PCR reagent was composed of 1× SYBR Premix Ex TaqII, 0.075 U µL^−1^ TaKaRa EX Taq, 0.6 mg mL^−1^ BSA (AS25483; AMEKO, China), 1 µM of forward and reverse primers, and 10^7^ to 10^5^ copies per µL DNA template.

The fluorescence detection unit that was assembled consisted of a 48W LED array (XPE60W, Cree, Durham, NC, USA), and a digital camera (Canon EOS 7D, Canon, Tokyo, Japan). A 480 nm narrowband filter was fixed in front of the LED array to provide the excitation light, and a 520 nm narrowband filter was fixed in front of the camera lens. The camera was connected to the laptop and automatically took a photo every 20 s. The fluorescent images were captured by the aforementioned digital camera, with parameters that were set as follows: F = 2.8, M = 1/20, and ISO = 2000. The ImageJ software was used to process the images and to distinguish the light from the surroundings. By calculating the light intensity of the liquid plug in each cycle, the fluorescent intensity curve of the PCR’s amplification could be obtained.

## 4. Results and Discussion 

### 4.1. Flow Analyses

#### 4.1.1. Results of the Experiment on Changing the Viscosity of the Oil Phase

**Figure 3:**
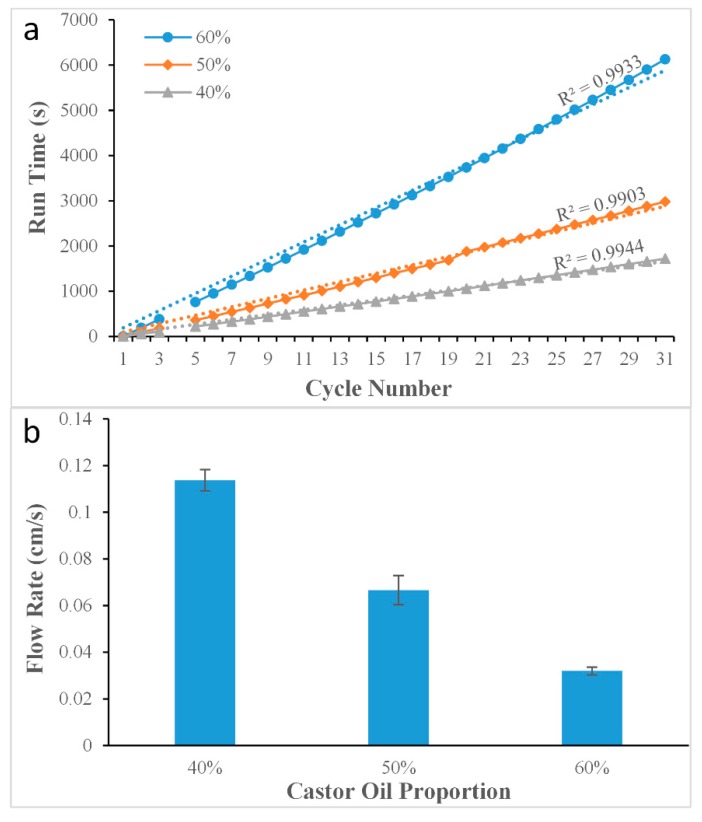
Results of experiment on changing viscosity of oil phase. (**a**) Flow time under different viscosity; (**b**) flow velocity under different viscosity.

Figure 3 has shown the effect of changing the viscosity of the oil mixture. The abscissa represents cycle number, which is the number of turns of Teflon tubes wrapped around the PDMS block. The temperature was set at 30 °C. The flow velocities of the liquids were stable when the tail Teflon tube (ID = 0.3 mm, OD = 0.6 mm) was stretched to four times its original length to 60 cm (ID = 0.16 mm, OD = 0.32 mm). The total run times of the liquid flowing inside the Teflon tube for the experiment were 6168 s, 2791 s, and 1545 s, when the viscosity of the oil mixtures was 60%, 50%, and 40% respectively. When the viscosity was 60%, the velocity of the liquid was extremely slow, and the situation was also similar when the viscosity was 50%. However, when the viscosity of the oil mixture was 40%, the flow velocity and the flow time per cycle were suitable for the PCR reaction, and the linearity of the curve was also better than the other two examples, and the $R^{2}$ value was calculated to be as high as 0.9944. The results suggested that the flow velocity of the liquid could be precisely controlled by changing the viscosity of the castor oil mixture.

#### 4.1.2. Results of the Experiment on Changing the Pressure in the Syringe

We connected the pressure gauge to the cavity of the syringe to measure the pressure of the compressed gas. During data acquisition, we used a video recorder to record the pressure when the liquid just enters the tube and then end the video recording when the liquid completely flows out of the thermal circulation tube. When collecting data points, we combined the pressure videos and the flow videos to extract data points and obtain pressure values.

**Figure 4:**
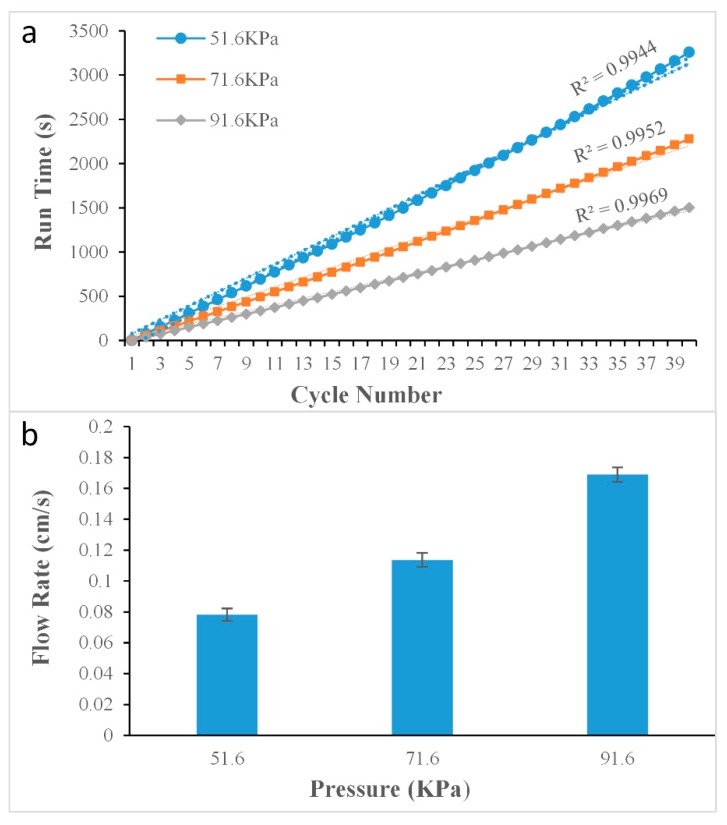
Results of experiment on changing pressure of the syringe. (**a**) Flow time under different pressure; (**b**) flow velocity under different pressure.

Figure 4 has shown the effect of pressure. We use a manometer to measure the internal pressure. It is connected under the syringe for measurement. The temperature was set to be 30 °C, the flow velocities of the liquids were stable when the tail Teflon tube (ID = 0.3 mm, OD = 0.6 mm) was stretched to four times its original size to 60 cm (ID = 0.16 mm, OD = 0.32 mm). The total run times of the liquid flowing inside the Teflon tube were 3233 s, 2283 s, and 1474 s, when the pressures were 51.6 kPa, 71.6 kPa, and 91.6 kPa respectively. When the pressure was 91.6 kPa, the flow velocity and the flow time per cycle were suitable for the PCR reaction, and the linearity of the curve was also better than the other two, and the $R^{2}$ value was calculated to be as high as 0.9969. The results suggested that the flow velocity of the liquid could be precisely controlled by changing the pressure.

#### 4.1.3. Holistic Analysis

**Figure 5:**
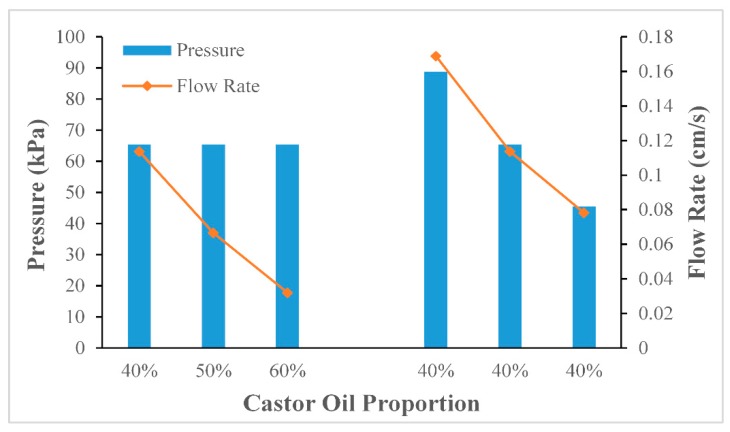
Average flow velocity under different viscosity (**left**) and average flow velocity under average pressure (**right**).

As can be seen in Figure 5, the data on the left side of the figure showed that by keeping the pressure unchanged, the flow velocity decreased rapidly when the mixing ratio of the oil mixture increased. It could also be seen that the data on the right side of the figure indicated that with the same viscosity, the flow velocity decreased rapidly when the pressure decreased. The previous two experiments showed that with the use of the Teflon tube that had been stretched to four times its original size, the system was able to achieve steady continuous flow. By adjusting the viscosity of the oil mixture or the pressure, the flow velocity and the flow time per cycle were able to be suitable for the PCR reaction.

### 4.2. Results for the Continuous Flow PCRs

**Figure 6:**
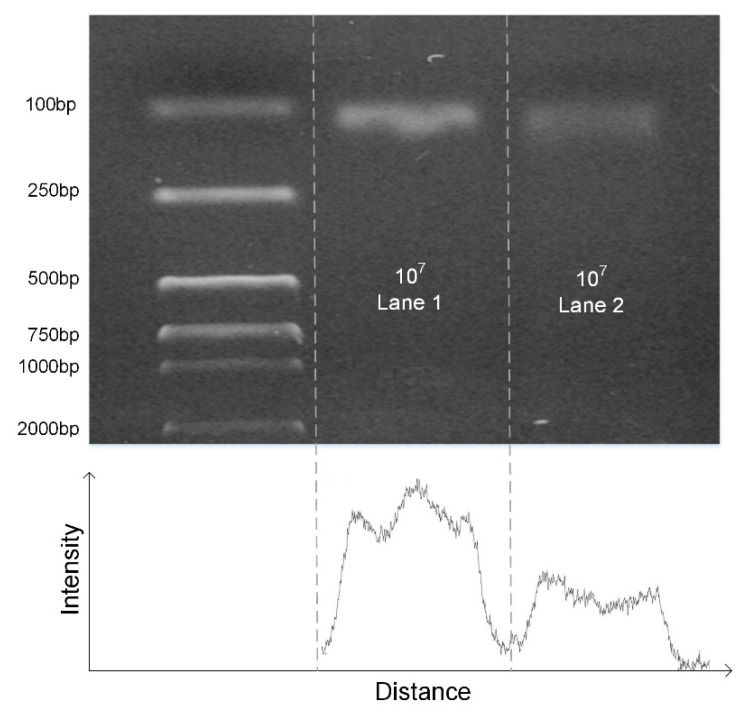
Electrophoretic results by commercial cycler (**lane 1**), electrophoretic results by microdevice (**lane 2**) and the ladder (**left**), and the intensity diagram of the polyacrylamide gel electrophoresis image.

A single heater was used to create thermal cycling for the PCR reaction. The PDMS block with the Teflon tube wrapped around it was placed on the single heater for approximately 30 minutes in order for it to reach the required temperature before the injection of the sample. The denaturation temperature was adjusted to approximately 95 ± 0.7 °C and the annealing temperature was adjusted to approximately 60 ± 0.8 °C. In order to ascertain the PCR amplification efficiency of the microdevice, the commercial quantitative PCR (qPCR) cycler (Bio Rad, Hercules, CA, USA) was used to setup a parallel experiment. The reaction conditions in the commercial qPCR cycler were setup as mentioned above, and the annealing stage and the denaturing stage were set at 35 s and 15 s, respectively. Meanwhile, the flow velocity of the reagent in the Teflon tube wrapped around the PDMS block was approximately 50 s per cycle. As shown in Figure 6, the two methods of the commercial qPCR cycler and the present system have the same components, and the amplification efficiency was similar. This means that the present system is fully capable to be used for the PCR reaction.

### 4.3. Results of the Real-Time Fluorescence Detection

For the real-time fluorescence detection, three serially ordered diluted genes with 10^7^ to 10^5^ copies per μL were tested in both the microdevice and the commercial qPCR cycler (Bio Rad, Hercules, CA, USA). The images were captured by a digital camera (Canon EOS 7D, Canon, Tokyo, Japan) and the intensity of the fluorescence of each cycle was analyzed using the ImageJ software.

**Figure 7:**
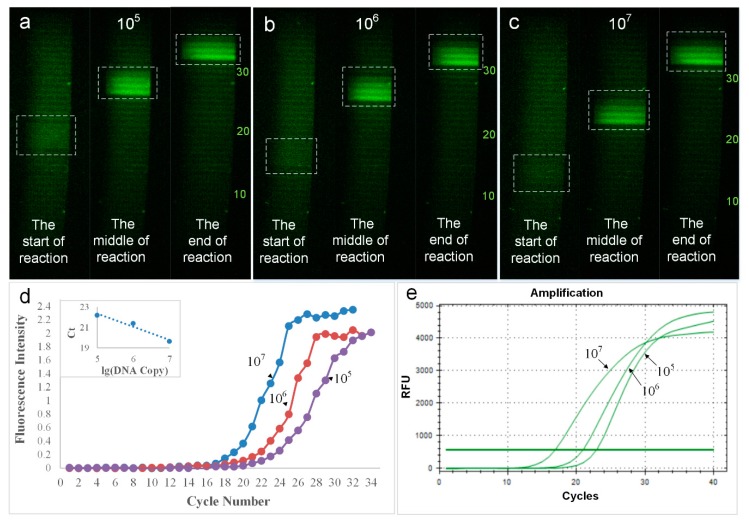
Changes of fluorescence intensity in PCR reaction under different DNA concentration (**a**–**c**); The amplification curves gained from the fluorescence images of serial diluted DNA molecules of the microdevice (**d**); amplification curves from the commercial real-time PCR amplification system (**e**).

Figure 7 has shown the change in the intensity of the fluorescence during the PCR reaction process. As the concentration of the DNA template decreased from 10^7^ to 10^5^ copies per μL, the number of cycles required to start the reaction increased. When the concentration of the DNA template was 10^7^ per μL, the reaction started on the 12^th^ lap, and when the concentration of the DNA template was 10^5^ per μL, the reaction started on the 18^th^ lap, which meant that the higher the concentration of the DNA template, the earlier the reaction started. The fluorescence intensity of the curves shown in Figure 7d were obtained using three serially ordered diluted genes from 10^7^ to 10^5^ copies per μL. The Ct values of the three curves were calculated to be 19.62, 21.38, and 22.19. The results produced by the commercial qPCR have been shown in Figure 7e, with Ct values of 16.92, 21.00, and 22.94. This suggested that the system could be applied to real-time PCRs, and the results were similar to commercial qPCR devices.

## 5. Conclusions

A novel mechanism for self-powered liquid transport in an end-open microchip has been introduced in this paper. It has provided a new approach to conveniently control the velocity of the liquid by stretching the tail Teflon tube to achieve the same effect as the previous methods but with no disadvantageous factors. The flow assays confirmed that the theoretical formula and the experimental results were well matched. Furthermore, by adjusting the viscosity of the oil mixture or the pressure in the syringe connected to the inlet, a totally original experimental assumption was set up with the previous mechanisms in order to affect the flow performance. In contrast with most other passive micropumps which have been previously developed, the passive micropump that has been developed in this paper displayed a superior pumping performance concerning its capability for use in complicated applications such as the home-made setup of continuous-flow real-time on-chip PCRs. In the future research, the system will be upgraded to make it more controllable, and to apply it to wider fields.

## Figures and Tables

**Table 1:** Viscosity under different conditions.

Condition	Classification	Temperature (°C)	Viscosity (mPa·s)
Mixture of Castor Oil (40%)	Experiment	30	21.7
Mixture of Castor Oil (50%)	Experiment	30	46.5
Mixture of Castor Oil (60%)	Experiment	30	57.4
Mixture of Castor Oil (40%)	Experiment	60	10.0
Mixture of Castor Oil (40%)	Experiment	90	5.4
Isopropyl Palmitate	Experiment	30	3.7
Isopropyl Palmitate	Theory	30	5
